# Conceptual scaffolding for the philosophy of medicine

**DOI:** 10.1007/s11019-024-10231-w

**Published:** 2024-10-28

**Authors:** Yael Friedman

**Affiliations:** The Centre for Philosophy and the Sciences (CPS), Department of Philosophy, Classics, History of Art and Ideas, University of Oslo, Oslo, Norway

**Keywords:** Pluralism, Disease concept, Epistemic injustice, Diagnosis, Wearables

## Abstract

This paper consists of two parts. In the first part, I will introduce a philosophical toolbox that I call ‘conceptual scaffolding,’ which helps to reflect holistically on phenomena and concepts. I situate this framework within the landscape of conceptual analysis and conceptual engineering, exemplified by the debate about the concept of disease. Within the framework of conceptual scaffolding, I develop the main idea of the paper, which is ‘the binocular model of plural medicine’, a holistic framework for analyzing medical concepts and phenomena. In the second part, I demonstrate the use and value of the binocular model by analyzing, through the lenses of the model, the phenomenon of health wearable devices and their effects on the concept of diagnosis.

## Part 1: Pluralism as a philosophical methodology

## Introduction

How can pluralism be used as a philosophical methodology? In the following, I will present ‘conceptual scaffolding’^1^, a philosophical tool to map a nonharmonic landscape of meanings. I will use the philosophical debate on the concept of disease to show how conceptual scaffolding relates to conceptual analysis and conceptual engineering, which are the common philosophical methodologies used in this debate. I will then introduce the binocular model of plural medicine as an example of conceptual scaffolding. I build the binocular model by developing, refining and integrating two existing central models of disease, and through this integration, novel dimensions of conceptual interactions are revealed.

The complexity of medicine, exacerbated by recent advancements in biological research and technology, underscores the need for a more systematic and pluralistic approach. New theories like epigenetics, process biology, and symbionts research are challenging traditional medical assumptions, emphasizing the intricate interplay of biological, environmental, and social factors that influence health and disease. Simultaneously, big data and immersive medical technologies are giving rise to new medical initiatives that increase the complexity of medical practice, requiring a broader range of perspectives and expertise. A systematic pluralistic approach would allow us to attend to the complex reality of medicine more easily.

Conceptual scaffolding is a method that allows us to acknowledge the different ways in which the same concepts are used by different actors, different fields of knowledge (scientific and non-scientific), and across different levels of analysis. It departs from an approach according to which part of a philosopher’s work is to bridge different debates by creating tools that accommodate the variety of perspectives and bodies of knowledge that are relevant for understanding a specific phenomenon or concept. In other words, conceptual scaffolding involves mapping and demarcating the landscape of uses and meanings from which the analysis and engineering work is performed. By scaffolding, I do not mean to suggest specific content for a concept but to suggest a framework under which one can develop models that help to delineate and track different meanings of a concept over time and across contexts.

I locate conceptual scaffolding as the step before the actual engineering of concepts and argue that it should be seen as a meaningful step that stands independently from the engineering of concepts. In that regard, conceptual scaffolding is not about arriving at the right or most economic definition of a concept (or a phenomenon). Differently from conceptual analysis and engineering, I am interested in systematizing dynamic frameworks that allow the plurality of definitions to flourish and reflect the network of relations in which they are understood and shaped over time. In that regard, scaffolding makes them more transparent and, by that, potentially allows for new philosophical insights to emerge.

## Philosophical methods in the debate on the concept of malady

To explain the utility of conceptual scaffolding, I will discuss it in the context of the philosophy of medicine. More specifically, I will use one of its most central debates on the concept of disease (from now on, I will refer to it more generally as ‘malady’) and place it in regard to the philosophical methodologies that have been used in its development.

While the classic debate on the concepts of malady and health employed *conceptual analysis* as its main methods^2^, some doubts about the usefulness of this approach have been raised in recent years. According to the philosopher Maël Lemoine (2013), the main goal of conceptual analysis is to examine a set of necessary and/or sufficient definitional criteria regarding specific phenomena and point out counter cases to existing definitions. In examining the usefulness of this method, he argues that conceptual analysis is a hopeless method to adopt if one wants to determine whether disease or health is a naturalistic or normative concept. Lemoine argues that conceptual analysis has an intrinsic limitation: “[I]f several definitions could match the same set of uncontroversial cases, it would not matter whether they agreed or disagreed on the controversial cases” (Hofmann and Svenaeus 2018., p. 316). Instead, he suggests *naturalizing the concept of disease* by looking at particular conditions and engaging more in experimental philosophy.

Similar to the philosopher Quill R. Kukla (2022b), I think that the debate between naturalism and normativism cannot be decided easily since the medical realm is messy and includes different uses of these concepts that cannot be dismissed, which renders the *pluralistic stance* in this case favorable. They write:

“In order to decide whether something should count as a disease, we need to answer questions such as: who exactly is helped and who is harmed by counting this as a disease? What are the social, ethical, and economic risks of bringing the group of people who have this condition under the surveillance and control of medical institutions? Will this classification serve the ends of science? Social justice? Economic justice? Will it stigmatize or destigmatize people with the condition? These are sometimes empirical questions that can be answered using the tools of psychology, economics, and anthropology, as well as biology. They are also often questions that call for normative reflection and reasoning. And the answers to these questions may not all line up the same way. There is just no reason to think that the answer as to what best serves economic justice is also the answer that will best serve science, and so on” (Owens & Alan Cribb 2019., p. 140).

In other words, if we want to have a singular concept of disease, we are forced to ignore a whole system of uses for different purposes.

Another (general) criticism of conceptual analysis comes from *conceptual engineers* (Cappelen 2018; Eklund 2014; Haslanger 2000). The philosopher Delia Belleri (2021) explains that “[t]he core ideas that motivate conceptual engineering are […] two. First, our concepts are not necessarily perfect, and may sometimes need improvements. Second, these improvements cannot be achieved *via* descriptive tasks of conceptual analysis; philosophers should, when possible, make a concerted effort to propose and advocate new, or revised, conceptual resources” (Owens & Alan Cribb 2019., pp. 1–2). Recently, the issue of pluralism became a topic of interest in conceptual engineering (see for example the contributions of: Belleri 2021; Dobler 2022; Nado 2021). According to Belleri (2021) there are different ways in which conceptual engineering could account for pluralism. My interest here is in pluralism regarding the different functions of concepts^3^, which together create a *conceptual family*, i.e., in which many meanings are encoded by one word (Dobler 2022, p. 2).

The philosopher Rachel Nado writes that “no single concept can offer ideal performance under such a workload” (Nado 2021, p. 2), so we need to allow a division of labor between the family members. According to Nado, we should revise our concepts by replacing them with multiple concepts (‘successors’). A different strategy for dealing with the multiple meaning of a concept, presented by Dobler (2022), does not see the multifunctionality as a deficiency that requires replacement. Instead, she suggests that in most cases it is enough that revision will be done by *‘calling to mind’* the different ways the concept can be used, and replacement might be used in cases of “specialised technical concepts that fulfill particular purposes” (Dobler 2022, p. 21).

The scaffolding work includes ‘calling to mind’ multiple perspectives, bodies of knowledge, and levels of analysis that are connected to a single concept. Conceptual scaffolding can be used to systematize these “callings” into organized and trackable models so that we can draw comparisons over time and between different contexts. The scaffolding models should be used to navigate the non-harmonious nature of the medical realm and could also be used as a tool for conducting better conceptual analysis and conceptual engineering (when possible or needed).

## Conceptual scaffolding of malady: two ancestor models

The two dominant conceptual models of maladies, on which I draw in building the binocular model, are the well-known biopsychosocial (BPS) model of medicine (Engel 1977) and the less-known but still highly important triad model of disease, illness, and sickness (Hofmann 2002, 2016a; Twaddle 1994a; Twaddle 1968). These models first appeared in the literature almost half a century ago and are still central to theoretical discussions on medicine today─ especially in philosophy, sociology, anthropology, and medicine-related education. Both models originally aimed to capture the full nature of maladies or unhealthy conditions (both ontologically and epistemologically) and are, therefore, relevant to consider and think through when developing a scaffolding model for the concept of malady.

The BPS model was presented by the internist and psychiatrist George L. Engel as the appropriate model for clinical work, research, and teaching in medicine (Engel 1977, 1978, 1980). In contrast to the biomedical model, the BPS model expands the spectrum of reasoning about maladies from only biological factors to include also social and psychological factors that contribute to one’s ill health (Engel 1977). The BPS is therefore a model that focuses on determining the cause of a malady, and how different factors play a role in it. In this sense it is a harmonious model, in which the biological, psychological and social factors (which can also overlap) complement each other in a larger causal picture. The BPS model’s disciplinary division has a strong ontological dimension: “The biomedical model embraces both reductionism, the philosophic view that complex phenomena are ultimately derived from a single primary principle, and mind-body dualism, the doctrine that separates the mental from the somatic” (Engel 1977, p. 130).

Although BPS is a dominant model, there is surprisingly no real biopsychosocial care in practice. While praised in medical education, the BPS model has also received much criticism over the years. Among these criticisms, it has been argued that the BPS model presents a problematic ontology; for example, it shows a misunderstanding of the idea of the dualism of mind and body (O’Leary 2021). It has also been accused of being too general a model and therefore as not being applicable to medical practice (Ghaemi 2009). Finally, while it gives account to the psychology of the patient, it has been argued that it lacks agential recognition (Bolton 2020; Bolton and Gillett 2019) and, therefore, does not fully acknowledge the patient as a person (Engel was an opponent of what he called the “humanistic dogma”).

From a pluralist perspective, the BPS model seems to provide an additive framework of evidence for why a patient has a poor health condition, rather than reflecting the dynamic nature of medical practice. But, putting its ontological premises aside, BPS does capture one essential dimension of medical realm in regard to maladies, namely that different kinds of knowledge are involved in medicine beyond biomedical knowledge. In the context of developing a scaffolding model, I suggest leaving the ontological commitments of BPS aside and moving away from the view that the medical realm is produced by empirical evidence from specific disciplines to see it as related to different bodies of knowledge: knowledge about biology, knowledge about the psyche, and knowledge about society, which is not tied to only one specific discipline (I will develop this idea in the section titled The binocular model of plural medicine).

However, this would still not be comprehensive enough for a scaffolding model, as there are not only different bodies of knowledge but also different perspectives that generate and perceive these bodies of knowledge. While Engel is interested in psychological and social knowledge, he considers it only from the perspective of the physician. Specifically, he diagnoses and evaluates a disease and sees the psychological and social condition of the patient on the basis of “stabilizing” and “destabilizing” factors (Engel 1980, p. 543) of the disease condition, without accounting for the patient’s perspective, the social perspective and the possible gaps between them and the physician’s perspective.

This brings us to the second influential model, the triad model of disease, illness, and sickness. This model was first introduced by the sociologist of health Andrew Twaddle in his doctoral dissertation defended in 1967 (Twaddle 1968) and revisited in 1994 (Twaddle 1994a, b), in dialogue with the philosopher Lennart Nordenflet (1994) and his critical standpoint. The definitions of the triad presented by Twaddle comprise both ontological and epistemological dimensions. From an ontological perspective, he suggested that disease is an organic phenomenon, illness is a subjective feeling, and sickness is an event defined and located in a social system (Hofmann 2002). From an epistemic perspective, disease can be objectively measured, illness “can only be directly observed by the subject and indirectly accessed through the individual’s reports” (Owens & Alan Cribb 2019., p. 653), and sickness is accessed by a failure to meet the social standard level of performance in society (ibid.). Twaddle saw the interconnection between these three and attributed a temporal (simplistic) dimension to the triad, in which a disease can become an illness and then sickness. One of the main criticisms of Twaddle’s definitions was formulated by the philosopher Lennart Nordenfelt in a published dialogue between the two (Nordenfelt 1994; Twaddle 1994b). According to Nordenfelt, the triad model cannot be useful without a general theory of health. Challenging Nordenfelt’s criticism of Twaddle’s definitions, the philosopher Bjørn Hofmann (2002) suggested a refined model, which set aside the ontological dimension and instead focused on the epistemic and normative dimensions of disease, illness and sickness.

This reformed triad model differentiates between three possible views on malady: the biomedical, the first-person, and the social. Hofmann defines disease as “a negative bodily occurrence as conceived of by the medical profession” (Hofmann 2002, p. 657). Next, he defines illness as “a negative bodily occurrence as conceived of by the person himself” (Owens & Alan Cribb 2019., p. 657). Finally, he defines sickness as “a negative bodily occurrence as conceived of by society and/or its institutions” (ibid.), which can also be framed by norms that can result in stigmatization and discrimination (Hofmann 2016a). Here again Hofmann emphasizes that the three concepts can overlap. He also notes that adoption of each perspective can change over time, as medical praxis can change and influence the definition of a disease, but the existence of the perspectives themselves remains. This more complex dimension of temporality is lacking both in the original triad model and in the BPS model, and is significant for a scaffolding model.

While the perspectivism suggested by Hofmann is very useful for a scaffolding model, the triad model does not account for how epistemic perspectives contribute and are affected by different bodies of knowledge, which one could describe as “objective” (this is not to say that the knowledge one obtains from different bodies represents the truth about the world, but that they are our best knowledge about the phenomena in question). This is where the BPS model meets the triad model.

Hofmann mentions that “[t]he social and psychological influences on the concept of disease are clearly reflected in the influential biopsychosocial model of disease” (Hofmann 2002, p. 666). He also writes that “the experience of illness is affected by medical knowledge” (ibid.) and later, in a paper from 2016 he adds that both illness and disease may be influenced by new imaging technologies (Hofmann 2016a). However, he also writes that the knowledge status of disease is objective, while the knowledge status of illness and sickness is subjective and inter-subjective, respectively. Putting these points together, it is rather unclear what the relations between the epistemic perspectives and the different bodies of knowledge actually are.

Differently from Hofmann, I suggest that perspectives are always produced from a specific position and, therefore, cannot be defined as objective. At the same time, subjective experiences and normative interactions can be collected and analyzed in a way that establishes objective knowledge on non-objective phenomena (Cunningham 2015). In other words, although the experience of an illness in an individual is subjective, we can also have research on patient experiences on this malady that constitutes a more objective body of knowledge about patient´s experiences. So, there are not only possible gaps between perspectives but also possible gaps between the different bodies of knowledge (e.g., psychology or phenomenology) and their related perspectives (first-person experience). In the same way, there are possible gaps between bioscientific knowledge on some case x and a specific doctor or a specific biomedical community perspective on case x.

This is all to say that, while both models fail in providing a clear ontology of the nature of malady, they both provide important ─ however partial ─ epistemological accounts for malady. To sum up, a scaffolding model that reflects the complex dynamics of malady and takes into consideration our shared world and situatedness should satisfy three criteria; First, it should include the different epistemic perspectives involved in the medical realm. Second, it should take into consideration the different bodies of knowledge that play a role in it, which I call ‘aspects’. Third, to capture the changes and exchanges between perspectives and aspects, such a model should include a temporal dimension. In other words, to capture the complications of the real-world concept of malady, it will be beneficial to bring refined versions of the BPS and the triad model together to enable the construction of more nuanced images of medical concepts. The binocular model, presented below is therefore not an entirely new model but a refinement, development and an epistemologically focused combination of the two accounts discussed, that bring forward novel conceptual dimensions.

## Medical scaffolding beyond malady

So far, I have discussed the criteria necessary for a scaffolding model of malady. However, I believe there is a need for a more general scaffolding model for medicine beyond malady that can serve as a tool to pursue nuanced critical analyses. In this respect, the motivation to introduce the model resonates with current trends in medicine that aim towards expanding its practical scope by drawing on more perspectives and more evidence from different areas of knowledge.

Two main trends in medicine can be mentioned here; First, big data and the new range of immersive medical technologies, such as health tracking devices, increase the amount as well as the different kinds of information involved in medical practice, opening the door to greater complexity. Navigating this complexity can be done in different ways. More types of data give rise to more views on maladies and more ways of observing and understanding the body. These technologies also create greater involvement from different actors in the medical realm and affect the current structure of medical practice, such as the doctor-patient relationship. There is, therefore, a growing need to disentangle the different facets of medical practice in order to better track what kinds of evidence are involved, who presents, produces, and analyzes them, and in what contexts and ways these actors and kinds of evidence interact.

Second, personalized and participatory medicine initiatives highlight the need to consider patient agency as a significant actor in medical decision-making and to tailor interventions that fit the patient as a person. In contrast to evidence-based medicine, these initiatives usually claim to set for themselves a broader aim of diminishing suffering and improving health and well-being, rather than “only” curing a disease (see, for example: Bittencourt 2018; Hanemaayer 2021; Vogt et al. 2016a). As a result, the patient’s view on their well-being and their social environment can be seen as carrying greater epistemological importance to medical practice. In other words, a more person-centered, individually tailored, and participatory medicine opens the field to additional perspectives. In particular, it requires a genuine incorporation of the patient’s perspective (Bardes 2012; Prainsack 2017). In addition, changing the focus from only curing a disease to preserving and promoting health and well-being emphasizes the need to analyze more aspects of life as relevant to medicine. This means that the medical field would be more occupied with psycho-behavioral, phenomenological, and socially related knowledge than it used to be.

The need for a comprehensive model is also growing from recent philosophical work that brings to the fore the context in which medicine is practiced^4^. The philosopher Anna Alexandrova (2017), for example, stresses the multiple faces of the concept of well-being by using the idea of *variantism*. According to this idea, there is no single theory of well-being that underpins the total number of constructs of well-being in life and science (Owens & Alan Cribb 2019., p. 27). A similar approach can be found in the work of the philosopher and ethnographer Annemarie Mol, who describes healthcare as an arena of different performing acts, which create various realities for the same phenomena. In her book *The Body Multiple* (Mol 2003), Mol fleshes out — both in content and form — the multiple views on the body through the case study of atherosclerosis. Although sharing a similar motivation to understand medicine as operating in the world, these contributions do not provide a systematic framework that encompasses the complex reality in which medicine operates, which can be used for further analysis.

Furthermore, methodological pluralism in medicine is also central to the work of the philosopher Miriam Solomon (2015). Solomon focuses on methodological pluralism in clinical research and clinical encounters. In other words, she inquires the plural ways in which medical knowledge is formed by analyzing the dynamics between different modi operandi used by the biomedical community, such as consensus conferences, evidence-based medicine, and clinical judgment, which can lead to opposing results. Her analysis includes considerations outside of the biomedical field, such as the preservation of medical authority in society or methodologies that include the patients’ view, but her focus is on pluralism that is internal to the biomedical community (what Bschir and Lohse (2022) call ‘intradisciplinary pluralism’). My focus on pluralism here is on pluralism “with science,” including knowledge and perspectives outside of the biomedical community and beyond the issue of methodology.

The scaffolding model introduced below, which I call the binocular model of plural medicine, is a comprehensive model, that can be applied to any medical concept, practice, or phenomenon, so it should not be concerned exclusively with the nature of malady. The suggested model presents a descriptive framework for mapping various perspectives and aspects regarding medical concepts, practices and phenomena and the interactions between the perspectives and aspects. It is not designed to take any normative stand, other than pluralism as an epistemic value, although it may prove helpful in making normative decisions on topics in medicine by providing needed nuance and mappings of existing complexities.

## The binocular model of plural medicine

### The model in brief

**Fig. 1:**
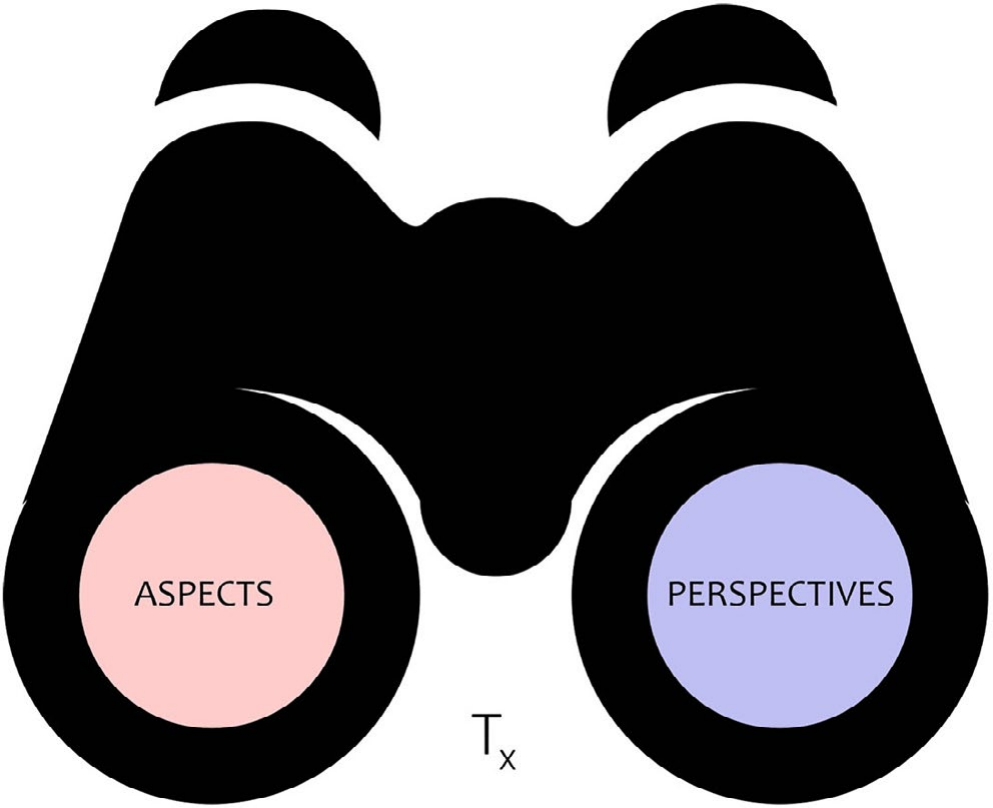
The Binocular Model

First, I give an overview of the binocular model of plural medicine before I provide a more in-depth presentation of the ideas involved. The binocular in the binocular model has two lenses: the lens of perspectives and the lens of aspects (Fig. 1). Viewing a concept or practice of medicine through the binoculars allows us to see the complex interactions between various perspectives and aspects. The perspectives lens focuses on the perspectives of individuals and groups and their situated viewpoints, such as the experience of a patient or the viewpoint of a medical practitioner. The aspects lens focuses on systems and bodies of knowledge that we may see as more objective, such as the knowledge taught to medical students about lung cancer or the system of laws and rules governing a societal practice.

**Fig. 2:**
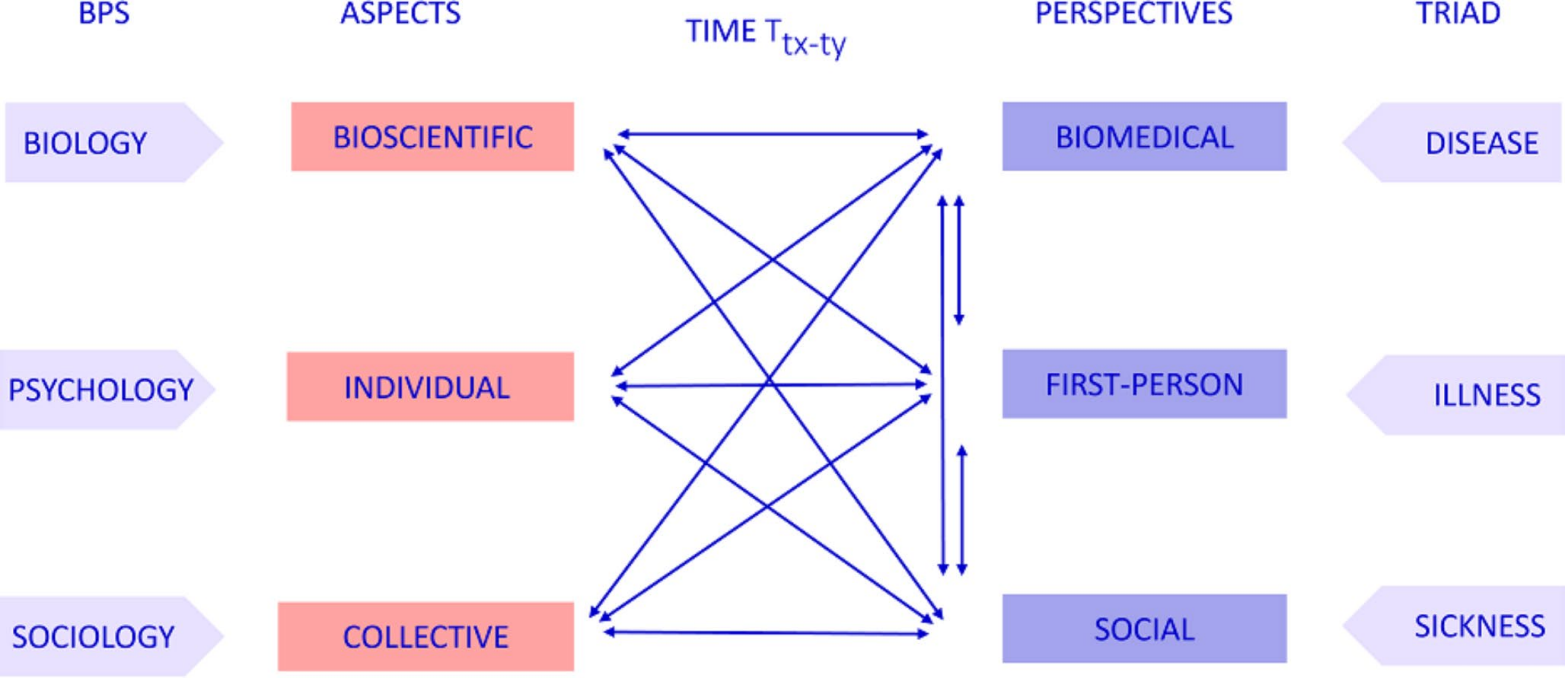
Aspects and perspectives of the binocular model, their interactions over time and their relation to the triad model and the Biopsychosocial model

Each lens can be understood as having three different “filters” that can also intersect with one another. The perspectives lens includes the biomedical perspective, the first-person perspective and the social perspectives while the aspects lens includes three groups of aspects: the bioscientific aspects, the individual aspects, and the collective aspects (Fig. 2). These aspects and perspectives are not exhaustive, and one can argue that more of them can be added; nevertheless, I take them as the key elements of this model.

To analyze a medical concept by use of the binocular model amounts to addressing the various aspects and perspectives connected to the concept and the interactions between them. For example, what the concept of ‘diagnosis’ signifies, will vary depending on which perspectives you emphasize (e.g. the patient´s, the doctor´s or the employer´s) and which aspects are in focus (e.g. research on patient experiences, biomedical knowledge, or laws regarding sick days). A mapping of the various interactions between perspectives and aspects will be valuable, for example when addressing questions such as who (or what) should decide a diagnosis? And how? Only after such a mapping, relevant actors could start answering the question of which perspectives and aspects should be given more or less weight.

In the scaffolding of a concept through the use of the binocular model, a more nuanced image may emerge of how the concept in question is used differently by different actors, in different fields of knowledge, and across different levels of analysis. Importantly, it shows how new dimensions of meaning come into view in the different interactive spaces revealed by the use of the model. As will be discussed in detail in part 2, we may recognize that the epistemic landscape of *diagnosing* is changing as patient perspectives, and the use of tracking technologies, are playing new and more important roles in diagnosis. As will be shown, the binocular model is of great help in tracking the relevant perspectives and aspects and their interactions regarding the concept of diagnosis, helping us to acknowledge the dimensions we need to consider for understanding diagnosis and for building a foundation for deciding how we should go about diagnosing.

### What is in its name?

Before delving into the details of the model, it is important to clarify its name. First, the term ‘Plural medicine’; plural medicine is a term I coin to describe the broad and non-hierarchical understanding of medicine as a holistic epistemic practice that involves *all aspects of life*, and in which the bioscientific perspective is not the only authority. By the term ‘plural medicine’, I refer to already existing developments of this kind as well as to an idea of medicine that is worth pursuing to a greater extent in the future. According to this view, medicine is understood as a practice that operates in the world, of which subjective and relational facets are integral.

Plural medicine does not assume pre-established epistemic hierarchies between different perspectives on medicine and medical practice. Rather, it seeks to acknowledge biomedical science, the reality as experienced by the individual patient, and the social outlook simultaneously and weigh their relevancy for decision-making on a case-to-case basis. Plural medicine does not deny the importance and strength of scientific evidence, but it does promote consideration of a range of perspectives and fields of knowledge that are part of the medical realm. Plural medicine should not be confused with either epistemic relativism (see: Chang 2012; Veigl 2021) or with pseudo-science. Inclusivity does not entail ‘anything goes’.

Second, I should explain the use of the metaphor of the binoculars. It is used to visualize the separation between aspects and perspectives central to the model that creates a more comprehensive picture of the medical realm (see below). The idea is that when looking through the binoculars with the two different lenses, a more holistic image emerges where you may see the various interactions between aspects and perspectives. This image provides a nuanced representation of various epistemic actors and their interrelations. We can use the binocular model to achieve a more refined understanding of any medical concepts, practice or phenomenon.

### Aspects and perspectives

This sub-section provides a more in-depth account of how I differentiate between the notions of aspects and perspectives. I take perspective, on the one hand, to refer to the view of an actor—this could be a single actor or a form of group agency. Perspectives are always situated and adopted from a specific position. Perspectives logically assume a plurality, since the medical reality necessarily contains more than a single perspective is able to capture. An epistemic perspectivism is therefore a form of labor division between different actors with different functions, experiences, and different kinds of access to experience.

Aspects, on the other hand, refer to the objects, the knowledge we have about the phenomena. Aspects contain different types of knowledge, such as factual, theoretical, conceptual, procedural and metacognitive knowledge, each of which relates to different scientific fields. A specific phenomenon can be captured by more than one aspect. Here I refer to three different groups of aspects that correspond to the disciplinary view of BPS (but not as exclusively as BPS does): *bioscientific aspects*,* individual aspects*, and *collective aspects* (see next subsection). A level of objectivity can be attributed to aspects (again, which is not equivalent to the truth), even if the knowledge in focus concerns subjective or normative matters (Cunningham 2015). For example, research on common feelings that arise in women in their 30s after chemotherapy for breast cancer belong to the individual aspect, while the laws on sickness benefits in a specific country belong to the collective aspects. This means that, while the focus of the bioscientific aspect lies on the objects of bioscience, i.e., biomedical knowledge (say, knowledge about the genetic nature of breast cancer), the focus of the biomedical perspective lies on the viewpoint of the individual physician or the biomedical community who observers the object. The biomedical perspective presented by, for example, a physician’s clinical judgment of how best to treat a biomedical condition (e.g., breast cancer), can differ from physician to physician, although they might be based on the same bioscientific knowledge.

### The model in more depth

The model suggests seeing aspects and perspectives in relation to each other and to the respective contexts at stake. It thus re-casts the discussion about health and medicine-related phenomena from a debate over factual issues to reflective analysis. The binocular model can be used to ask whether a perspective or an aspect are present or not in a specific context, inquire about their content and significance and follow how different perspectives and aspects affect each other. The model invites different levels of analysis. The analysis could be local, global, or universal, being undertaken at different degrees of specificity or generalization. For example, it can be used to look at a specific case of a patient or a specific health condition, focusing on a group of patients with the same condition or, more generally, on the ill. It can track a specific disease over time or implementation of a specific technology and its effects on different aspects and perspectives. It can also give us a picture of the dynamics between the observed (aspects) and the observer (perspective) and how it affects the concept/phenomenon in question.

Temporality also play a significant part in the model. Looking through the binoculars allows us to see a comprehensive picture of the medical realm regarding a specific context at time T_X_ or time period Ttx-ty. Temporality can have two meanings here: the first is the time in which one conducts the analysis using the binoculars, i.e., the time of the observation, while the second is the period of time which one is interested in analyzing. The temporal dimension allows us to compare the same context at different points in time (Tx, Ty) and track the relations between the model elements and the changes they make in the picture. It not only includes the development of scientific knowledge (through, for example, epistemic iteration), norms, and beliefs, but can also be used to highlight the diffusion and exchanges that might happen between the different elements (e.g., the diffusion that can happen between the bioscientific aspects and the first-person perspective). In this sense, the analysis can also account for Ttx-ty.

Let us start to unpack the different aspects and perspectives. The bioscientific aspects comprise the scientific biomedical corpus of knowledge that is produced by different scientific methods. I use the term ‘bioscientific aspects’ and not ‘biomedical aspects’ because it includes related scientific fields that contribute to biomedical knowledge (take, for example, how aerosol science can contribute to knowledge about infectious disease). The biomedical perspective, in contrast, represents the view of individual practitioners, healthcare providers, and the biomedical community’s group agency^5^. While the group agency of the biomedical community should align with the biomedical knowledge, it does not perfectly align with the perspectives of the individuals who comprise this group. Different biomedical communities can disagree on medical issues or methods on which there is no consensus (Solomon 2015), or simply have different thought styles (Fleck 1986b). A small biomedical community can still hold beliefs that the larger biomedical community rejects, such as in the classical case of peptic ulcer (see: Cherry 2002). Furthermore, since the biomedical community is part of the general community, there is always a small but nevertheless potential risk of bias. The biomedical perspective is not always portrayed as ‘a view from somewhere’, as the first-person or the social perspectives are, and is often understood as objective. However, the biomedical community is not free from bias, as race-based biases (see for example: Hamed et al. 2022), and gender-based biases (see for example: Younas 2021) do arise (among others). As the sociologist of medicine Nikolas Rose puts it, we often “ignore the fact that stigma and discrimination do not only exist ‘in the general public,’ but also among health care practitioners” (Rose 2018, p. 171). Biased biomedical views can easily be recognized retrospectively in light of updated biomedical knowledge (e.g., the cases of drapetomania or homosexuality).

The individual aspects comprise knowledge as studied by psychology and the behavioral sciences as well as the body of knowledge of phenomenology. Individual aspects also include knowledge about the experiences of illness as determined by the analysis of patient interviews, testimonies, or knowledge collected through patient organizations. In other words, it is the embodied knowledge of the patient, the ‘know-how’ of being ill (or healthy/well/recovered, etc.), that can be collected, analyzed and shared (see: Pols 2012; Ryle 1945). The term ‘individual aspects’ is used instead of ‘psychological aspects’ to indicate that it includes more than psychology (BPS) but does not dismiss psychology by using ‘phenomenological aspects’ (which can also be confusing, since it is often not distinguished from the first-person perspective)^6^. The first-person perspective^7^ of any given patient on a given occasion of salience, on the other hand, is unique and cannot be expected to capture completely the individual aspects. It does not capture them through psychology or behavioral science, and even research on the “phenomenology of” a medical issue (e.g., cancer) is limited in the way that it does not necessarily fully capture the peculiar private experience of a specific individual (but rather describes a phenomenon at a higher level of abstraction). While the way to deepen our knowledge of the experience of illness depends on gaining more understanding of individuals’ experiences, there will always be more to an individual’s first-person experience than our knowledge can reach. This non-alignment and the difference in accessibility^8^ between aspect and perspective is an important reason why empathy and humility have such a crucial role in clinical work. This difference between the first-person perspective and the individual aspects also marks a difference from the BPS model, which focuses exclusively on aspects, and the triad model, which focuses on experience^9^.

Lastly, there are the collective aspects and the social perspective. The collective aspects include societal practices, laws, policy, and regulations of a specific society, for example laws regarding sick leave, health benefits, or social security. Although “politics makes policy” (Jasanoff et al. 2021, p. 28) in democratic societies, the design of laws, policy, and regulations also involves knowledge from other scientific fields (Bschir and Lohse 2022), such as economics and social science, which are therefore part of the collective aspects^10^. These sociological and even bureaucratic dimensions are often neglected in medical epistemology, but they are relevant when it comes to understanding changes to a social context, as in the case of migration, for example, or within the social context, as in the case of a pandemic. On this global level, universal ethical principles and human rights also form essential parts of the collective aspects relevant for medical practice and human dignity^11^.

The social perspective includes the belief-systems of actors and institutions in society that shape behavior and laws. Similar to the biomedical perspective, it encompasses both individual and collective dimensions. In other words, individuals and smaller collectives (such as a family, work cohort or interest group) can have different perspectives on the same matter. Also, a particular society can have a perspective deviating from universal ethics and laws, leading them to behave differently than expected, following goals not regarded as beneficial (and ethical) to the society in large (locally or globally). In this sense, although individuals’ perspectives form the sociological norms, behaviors and laws, they are not equivalent and can even contradict each other.

As in the BPS model, the aspects of the binocular model have intersection points. For example, neuroscientific knowledge can be understood as an intersection between the individual and the bioscientific aspects. Public health knowledge can be seen as an intersection between bioscientific and collective aspects. Psychiatric and psychological conditions that relate to social interaction, such as agoraphobia and depression, could be understood from the intersection of three aspects or only the individual and the collective aspects (depending on the case and the coping strategies at hand).

Like aspects, and like perspectives in the triad model, the perspectives in the suggested model also intersect. Patients and the biomedical community are part of the society at large. This means that in some context they can share perspectives, for example in cases where biomedical knowledge is reasonably well embedded in society, as in the case of knowledge about basic hygiene. In addition, individuals that are part of the biomedical community are also patients in other contexts. When a medical expert self-diagnoses herself, one gets a first-person biomedical perspective and a first-person patient perspective at the same time. The problems do not arise so much at the intersections but where two or more perspectives collide and present conflicting evidence. This can happen, for example, when considering whether one is recovered or not (Friedman 2021). A patient can be recovered biomedically so the biomedical community would no longer see the patient as being in need of medical attention (biomedical perspective). However, the patient can still feel ill (first-person perspective) and troubled. Such a situation could arise after experiencing a life-threatening crisis (e.g., cancer or myocardial infarction) and may require a different kind of treatment on a different time scale.

Last but not least, there are also intersections between aspects and perspectives. One example is how the general public and individuals internalize biomedical knowledge in a way that affects their perspectives. Some medical terminology has become the *lingua franca* to describe a suffering experience. Health literacy is an obvious arena where biomedical knowledge is acquired to affect people’s attention and behavior (Kickbusch et al. 2013). Biomedical knowledge affects how a person describes their experience (Laforest 2020) and can increase or decrease their fears regarding a specific phenomenon that was not part of one’s experience before acquiring this knowledge (Watters 2010). The intersections between aspects and perspectives are, therefore, dynamic and changing over time.

### Limitations of the binocular metaphor

It is important to bring to the fore some limitations of the binocular metaphor. When looking through an actual set of binoculars, the picture one sees is a result of the alignment of the view (the shared field) from both lenses by the brain. However, in the model case, the picture one gets is not a result of an alignment of the two pictures but rather a combination of them. In fact, the model is based on the idea that perspectives cannot be identified with aspects. Thus, the “depth” of the analytical picture produced by the binocular model arises from the non-alignment of the different views from the two lenses. This non-alignment is where the binocular model differs from the narrow causal harmoniousness of the BPS model and the implicit harmoniousness in identifying the biomedical perspective and biomedical knowledge in the triad model. In the binocular model, the observer also gets one picture, but it is a multi-faced picture that can portray gaps and mismatches and not a monocular one, as in the BPS model. That is to say, the failure of the binocular metaphor is the same failure of the harmonious picture; the perceptual metaphor collapses when meeting the complex medical realm. I take this failure to be an essential component of conceptual scaffolding, which aims to hold contradictions and provide a holistic picture that is inherently nonharmonic. Therefore, the model’s name is a reminder of epistemic humility.

## Concluding remarks

The binocular model is designed to cover and reveal the complexity of the medical realm by differentiating aspects and perspectives and to be used to process interests and effects in a comprehensive, non-hierarchical way. It allows us to increase awareness for whether a medical test, tool, protocol, practice, phenomenon, or concept serves the individual, the case and/or the society. The pluralism intrinsic to the binocular model is designed to provide an analytical framework to examine epistemic dimensions and, thereby, the ambivalences of medical phenomena in today’s complex reality. It hence embraces pluralism as an epistemic value without subscribing to a specific ethical framework.

## Part 2: “Diagnosing” ‘diagnosis’ through health-tracking wearable technologies

## Introduction

In the following, I will use the binocular model of plural medicine to conceptually scaffold the concept of diagnosis in the context of health wearable devices. This part of the article will demonstrate how the dynamics between aspects and perspectives can contribute to philosophical analysis, exemplified here in the context of the philosophy of medicine.

In the next sections I will introduce the concept of diagnosis and health wearable technologies. The section titled “Wearables and the concept of diagnosis” is dedicated to the conceptual scaffolding. The last section concludes by showing how the division of labor between the different actors in the medical realm is changing due to the implementation of wearables, and what are the possible epistemic implications of such a change.

## Diagnosis

Diagnosis is a key medical practice that guides medical decision-making. Diagnosis usually refers to the biomedical identification of diseases. By determining how healthy and unhealthy states are differentiated, diagnosis directs what kind of treatment a patient should receive (or not receive); it serves as the epistemic hinge for decision-making, and, therefore, requires special attention. The philosophical discourse on medical diagnosis is mainly centered around the issues of methodology and evidence (e.g., Stanley and Campos 2013), which are usually understood as the signs and symptoms that a patient exhibits. As such, in philosophy, discussions of diagnosis regard issues like measurements, validity, utility, and consensus (see, for example, First 2010; Kendell and Jablensky 2003; Kendler and Solomon 2016; Solomon 2015)^12^, as well as the “logic” of diagnosis (Sadegh-Zadeh 2012; Upshur 2005). A recent book by philosopher Ashley Graham Kennedy looks at diagnosis more closely, and discusses diagnostic reasoning and testing in regard to not only evidence but also ethics and justice (Graham Kennedy 2021b). In addition, she discusses the issues of overdiagnosis and underdiagnosis (Graham Kennedy 2021a). These issues are also of special interest in the philosophy of medicine, and more so in recent years when fast technological developments enabling early detection and prediction and, at the same time generate new diseases and creates many false alarms with both ethical and epistemological consequences (Hofmann 2016b; Hofmann and Welch 2017; Vogt et al. 2019; Walker and Rogers 2017). Separating it from many other scientific practices, diagnosis contains not only elements of knowledge but also attributions to specific case classifications. Diagnosis intrinsically involves legal deliberations about potential actions, ethical considerations and normative decisions. Attributing a diagnosis is usually seen as a predominantly biomedical decision made by a medical professional and which can be debated among professionals.

Etymologically, the term ‘diagnosis’ comes from the conjunction of two Greek words: *dia* ‘through’ and *gignōskein* ‘recognize, know’ (“Diagnosis,” n.d.). “Recognizing thoroughly” is also what I aim to do with the concept of diagnosis itself in the following discussion. More specifically, I will conduct a conceptual scaffolding to the concept of diagnosis through the case of health-tracking wearable technologies using the binocular^13^ model.

## What are wearables?

Wearables are used with the aim of monitoring known health conditions (especially in the context of chronic conditions), preventing unknown conditions and improving well-being. In particular, I focus on wearable technologies that are used to track a person’s state of health, and it excludes other wearables that can be used in medical contexts, such as virtual reality glasses or pain simulators.

Wearables are situated at the intersection between tracking technologies, generally conceived, and telehealth (health from a distance). The former includes different tracking tools that are not necessarily wearable, such as monitoring apps, sensors, and cameras. The latter includes telecare, which is focused on patient-professional caregiver relations, and telemedicine, which focuses on professional health institutional relations (Pols 2012). Many wearables are connected to digital platforms, in which the data is shared automatically or voluntarily. Digital platforms are, for example, communication platforms with medical care that receive data from wearables, and dedicated forums and social networks. Examples of that are the platform associated with FitBit and Ultrahuman wearables. I am especially interested in wearables that extend beyond their hardware and include digital platforms. Wearable devices come in different forms, such as watches, patches, badges, bracelets, and similar products that are equipped with tracking sensors.

The data monitored by wearables are diverse and can include heart rate, blood saturation, body temperature, movement, sleep, EEG, menstrual cycle, and other biomedical parameters. In addition, wearables can monitor social interactions and translate them into biomarkers, such as voice tracking biomarkers that can help to detect depression (e.g., Loftness et al. 2023; Sheikh et al. 2021; Wolf 2009). In a sense, tracking technologies are not new; people have been tracking themselves using journals and diaries, creating protocols of how they feel, when, where, and at what rate they experience pain, and tracking their diet, weight, and pulse, depending on the context of their health issue. However, there is a difference between data entry and autonomous technologies with surveillant characteristics in the scope and amount of data that can be followed and analyzed.

Nor are health measuring tools new. Fitbit and Apple watches can be seen as a contemporary evolution of Sir John Floyer’s first commercial pulse watch that was available already at the beginning of the eighteenth century. However, the novelty of wearables lies in the amount of data they register and the constant tracking mode, the new kind of measurement they offer, and the availability of measurements that were not easily accessible to the user before (if at all). In some cases, the wearables also provide connectivity to the healthcare professional, where, in the case of deviation from the user’s normal measurements, the information is immediately delivered for professional assessment. A study on COVID-19 detection using Apple watches and a custom Warrior Watch Study app, is one example for such a use (Hirten et al. 2021).

The increasing use of wearables affects the question of who makes the diagnosis or, in other words, from whose perspective the diagnosis is made. Although wearables are not necessarily introduced as a diagnostic tool, they serve as such in practice. For example, a wearable watch that follows one’s heartbeat might not aim at diagnosing myocardial infarction. Still, it diagnoses in practice by automatically calling out an ambulance in case of a suspected heartbeat data pattern that fits with a typical myocardial infarction heartbeat pattern.

## Wearables and the concept of diagnosis

Under the pluralistic conceptual umbrella of malady, different actors produce different knowledge about a mal condition: the biomedical community produces knowledge about a disease, the patient (first-person perspective) produces knowledge about an illness, and society produces knowledge about a sickness. Some of the knowledge produced by each actor’s perspective is then shared and used to inform the other. For example, the symptoms experienced and reported by the patients and accumulated into what I call in the model ‘individual knowledge’ help the biomedical community produce knowledge about the disease. Another example is where knowledge about a disease is shared with society in order to take safety measures. Take, for example, how first-person experience of fatigue and fogginess post COVID-19, accumulated into individual knowledge on post COVID experience of symptoms that then helped the biomedical community to gain more knowledge about what is now called Long COVID, and how that affected changes regarding sick leave and social attitude towards the patients (HHS 2021; SOM 2022). In this case, we can say that we gained a shared perspective about a condition over time^14^. However, as explained in part 1, the different perspectives do not always go hand in hand, and gaps between perspectives and aspects can be observed.

**Fig. 3:**
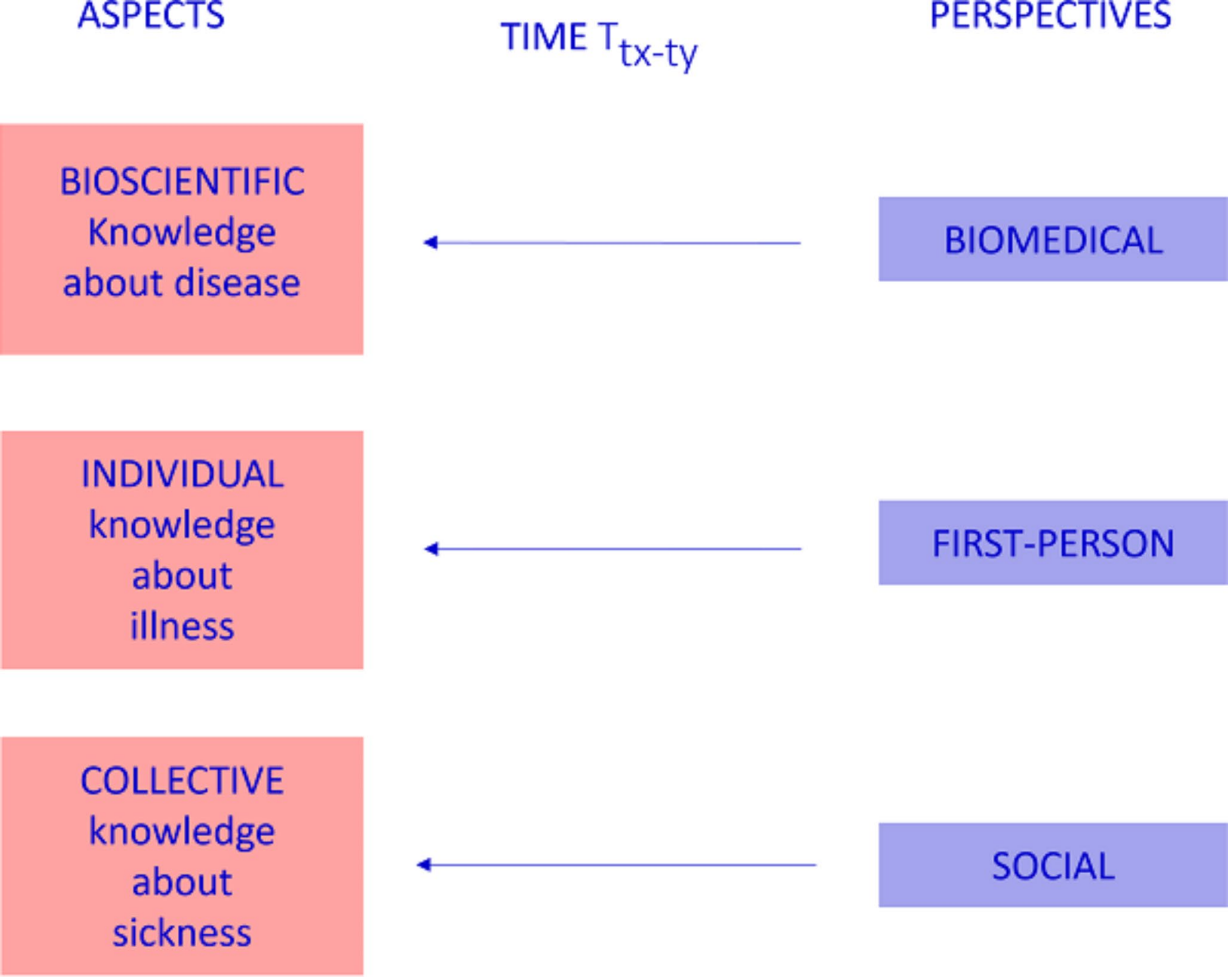
The traditional dynamics of diagnostic processes through the binocular model

Similarly, a pluralistic view on diagnosis as an umbrella term will include under the umbrella not only the diagnosis of a disease, but also the diagnosis of a person’s illness and sickness^15^. Thus, my basic assumption is that diagnosis is not a single event (diagnostic process) made by the biomedical community, but a series of different events (diagnostic processes) related to three viewpoints and aspects (Fig. 3). Here, I am interested in the division of labor that is involved in recognizing disease, illness, and sickness. In the following, I will focus more specifically on the case of wearable health technologies, asking whether and how wearables affect this division of labor.

**Fig. 4:**
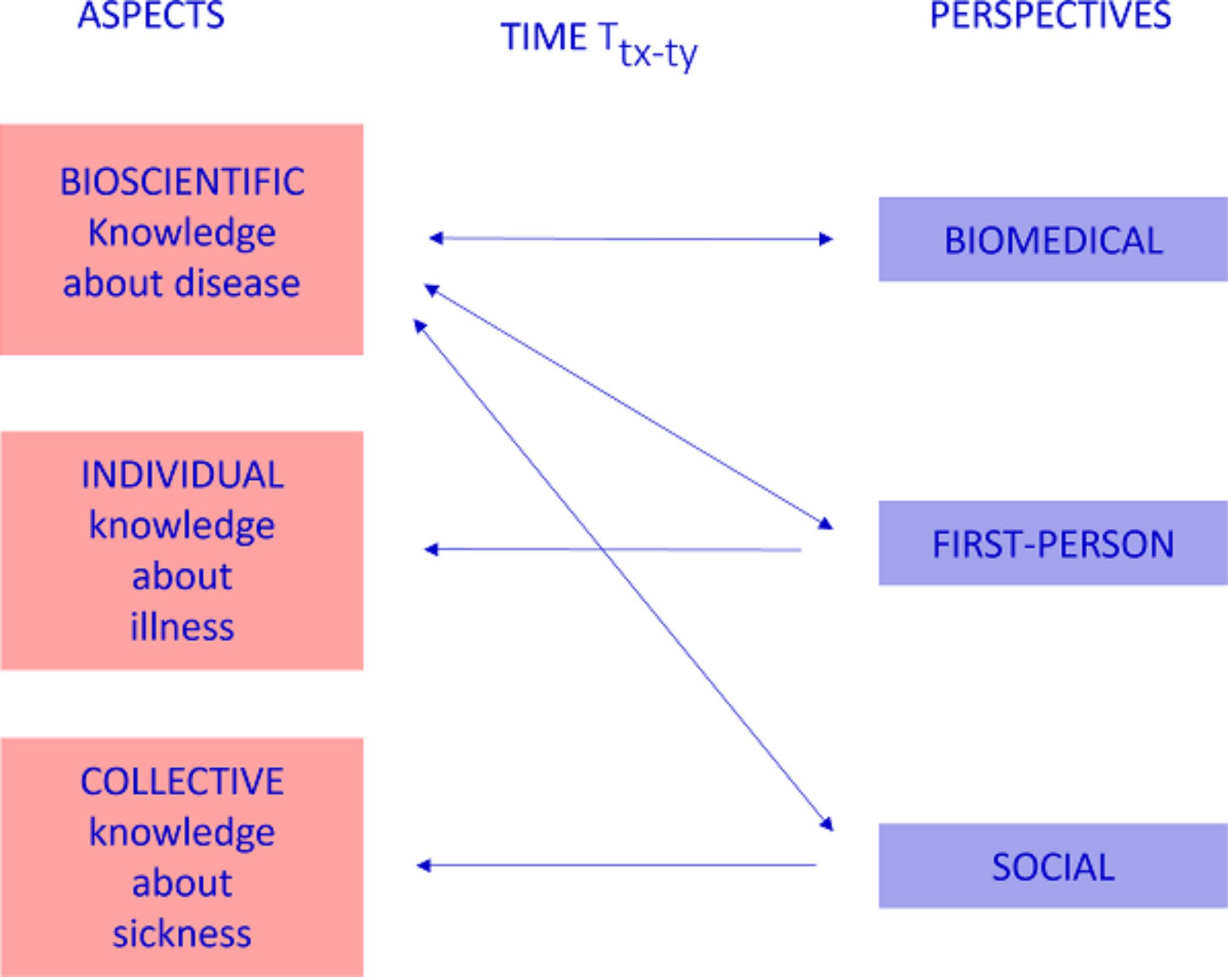
The dynamics of diagnostic processes using wearables through the binocular model

Using the binocular model’s perspectives and aspects division, I argue that wearables change the traditional division of labor of diagnosis. While there are many new medical technologies that affect diagnosis, wearables are unique as they lay in the intersection between medical technologies based on big data and popular medical commodities. In addition, the rationale behind wearable technologies that are used for tracking individuals’ health is also a feature shared with the P-medicine movement (e.g., increasing patient participation, personalization, and prevention) and can be conceived as part of its technological toolkit^16^. This combination enables a new dynamic of diagnosis, in which all actors diagnose a disease (Fig. 4). The separation between aspects and perspective is helpful here as it allows us to see how the relations between the different actors and knowledge production changes when we include biomedical data under bioscientific knowledge.

### Biomedical perspective and bioscientific knowledge

The biomedical perspective can be affected by measuring instruments and by new evidence acquired by them (see Fleck 1986a). Data patterns create new diseases biomarkers in areas where they did not exist before and affect diseases nosology (Vogt, Hofmann, & Getz 2016a; Vogt et al. 2016b). In addition, wearables affect also the traditional separation between normal and pathological, leading to medicalization of conditions that were considered healthy before (Hofmann and Svenaeus 2018; Hofmann and Welch 2017). This may leave overdiagnosis as a meaningless term because nothing is significant when everything is significant (Hofmann 2016b). The data is accepted by individual actors of the biomedical community to fit with the scientific system of knowledge. So, just like any other medical instrument, wearables can affect the biomedical perspective.

In this regard, wearables raise similar concerns to those which arise around P-medicine and other medical technologies based on algorithms and big data. More specifically, there is a concern that while the biomedical view incorporate new actors like IT companies, health professionals do not hold the technical understanding required to critically assess the validity of the algorithms that constitute these technologies (Christiansen 2020) which create a fragmented landscape of evidence based on big data that raise many uncertainties (Kimmelman and Tannock 2018; Lohse 2023). The current lack of literacy might also have long-term consequences as biased algorithms create biased data that preserve and aggravate the biases (see, for example, Hanna et al. 2020; Obermeyer et al. 2019).^17^

One can argue that wearables are just like other new diagnostic technologies based on algorithms and big data. However, new diagnostic technologies (like algorithms-based imaging technologies) are operated by biomedical professionals in a clinical setting, whereas wearables are operated by the users (patients) themselves. The users often lack bioscientific literacy and differ in their level of digital literacy. While health professionals would not necessarily know how the algorithms work, they have proficiency in using the tools correctly and the bioscientific knowledge needed to read their outputs critically.

In addition, some of the wearables that are available for users are technologies that improve well-being more generally (in contrast with, e.g., medically approved saturation measuring devices or drug pumps), e.g., by improving sleep or fitness. The concerns about them are similar to those regarding supplements and diet products that are propagated as healthy by interested stakeholders while some of them are bypassing basic scientific research. In other words, the biomedical perspective is not only enhanced by actors that are not part of the biomedical community, but, in some cases, *these actors also impersonate the biomedical perspective*.

While new medical instruments can be understood as shaping scientific facts (Fleck 1986a), they can also directly shape the first-person and social perspectives and influence the diagnosis made by these actors. Impersonation of the biomedical perspective can also lead patients to diverse or conflicting treatment paths when patients do not necessarily turn to the healthcare system that could potentially (and eventually) become more effective. This makes patients potentially more susceptible to pseudo-science and harmful treatments and contributes to the spread of misinformation, which affects the confidence of the public in biomedical experts. That is to say, the enhancement of what is considered a biomedical perspective by wearables that collect and analyze bioscientific data might lead to decreasing trust in biomedical experts^18^, which is a form of epistemic injustice. So, users’ trust in the data does not reflect their trust in biomedical experts (biomedical perspective).

### First person perspective and bioscientific knowledge

Diagnosis from the first-person perspective usually contributes to knowledge about illness. From *the first-person perspective*, wearables carry the promise of enhancing the user’s autonomy and can help to increase patients’ participation and decrease epistemic injustice. The act of self-diagnosis based on empirical data can be liberating, at least when the data matches the patient’s bodily experience. This is especially true for individuals whose self-testimonies are undermined or overlooked by health professionals. Among the more exposed groups are immigrants (Castañeda, 2022), children (Carel & Györffy, 2014), patients with psychiatric-somatic comorbidity (Bueter, 2021), and the disabled (Peña-Guzmán and Reynolds 2019), to name but a few. For these people, wearables can provide new opportunities to play a more active role in medical decision-making, act meaningfully^19^ to acquire better care and improve their health condition^20^,^21^.

However, with the advancement of medical technologies and enhancement of medical knowledge the experience of symptoms is not needed for the diagnosis of diseases (or pre disease, or risk to develop a disease, which are in many cases treated as if there is an actual disease). This phenomenon is discussed in a recent paper by the phenomenologist Fredrik Svenaeus (2023) as the objectification of the users. According to Svenaeus, this objectification can lead to either enhancement of the bodily presence or self-alienation.

In a sense this is not a new phenomenon. Early detection tests (e.g., for cancer), create such objectification without the patient experiencing any symptoms. However, these tests are not considered self-diagnosis. Self-diagnosis tests, like home kits to detect Influenza, also differ from wearables; besides high waves, they are not used on a regular basis. The diagnosis of Influenza is an event, not a continuous tracking. And while an Influenza test can change the way a person perceives their body (especially if they are asymptomatic), this change is temporary, and not extending over a long period of time like in the case of wearables. Lastly, wearables are also used to improve well-being, so their effect on perception is not limited to a disease state.

Wearables require individuals to self-locate themselves in terms of their ‘quantified self’ (Lupton 2016a, b). In other words, they should see how their own embodied experience stands regarding their collected individual data. This self-location sometimes involves ‘fitting process’ (Pols 2012) between the way that individuals feel (the first-person perspective) and what the data indicate that they should feel (bioscientific aspect), and it can include ‘self-doubt’ or ‘data doubts’ (Lomborg et al. 2020).

In their research on heart patients using Fitbit wristbands for self-tracking, the communication scholar Stine Lomborg together with colleagues from STS and data studies interviewed patients who self-locate themselves differently regarding their data (Lomborg et al. 2020). According to their report, there are three kinds of patients that belong to two different groups. The first group includes patients who do not perceive themselves as ill and used FitBit as a self-nudging wellness tracker. The second group includes patients who see some relation between the Fitbit data and their illness. Here, there are two kinds of patients: some will hope that the tracker will help them become well and will use it for emotional comfort, whereas for others the use of wearables only creates new worries and anxiety.

Lomborg and colleagues’ research shows that the way that one feels about one’s own condition can have an influence on how one interprets the data. In other words, the first-person perspective affects how the bioscientific aspects (and the individual aspects, now intersect to a greater degree with the bioscientific) are read by the patient. It can then also predict whether a wearable device can help or worsen an individual’s condition and if other diagnostic methods should be used instead (a ‘meta’-individual aspect). So similar to Svenaeus (2023), it shows that the quantified self can trouble the embodied feeling of one’s own body, which is in many cases the first basic indication one has that something is wrong (the bioscientific aspects affect the first-person perspective).

One can argue that the construction of people’s own narrative is already done through biomedical narratives, and the case of wearables holds no difference. While the first part is true (see, for example: Laforest 2020), wearables are unique in the sense that they can alienate the person from their body to a greater degree, since the use of wearables does not necessarily originate from an ill feeling and the alienation could be for long periods of time. While we use to think of the effect of biomedical narratives and imaging technologies as providing metaphors and ways of expression^22^, wearables not only affect how user describes her narrative, but construct her a new narrative; according to Hofmann and Svenaeus (2018, p. 7) “technology shapes our illness experience through the social norms and values fostered by technology” (Owens & Alan Cribb 2019, p. 7), and can “change symptoms formation” (Owens & Alan Cribb 2019., p. 5) and “even replace the experience of illness” (Owens & Alan Cribb 2019., p. 4).

Continuing Hofmann and Svenaeus points, one can say that wearables bring to the emergence of both illness and disease and that illness knowledge can be partially or fully replaced by data narrative of disease. This is not because wearables affect the organism to develop a disease (although stress as a result of the exposure to the data can potentially do that too), but because the acceptance of the narrative suggested by the data as evidence. Irregular data patterns translate into diagnoses like irregular heartbeat or sleep apnea. The self-diagnosis leads to illness, but the ill feeling originates from without, from the data, and not from within, from the person. Sharing this ill feeling on associated platforms or with a medical caregiver does not necessarily mean sharing experienceable knowledge only the person can access, but sharing the narrative constructed and conducted by the wearables.

In addition, as a popular commodity with associated communication platforms, wearables relate to the overarching theme of how life in a society interacts with and affects human health and suffering. As such, wearables also share similarities with other medical commodities and information sources (like the internet) involving patients’ self-diagnosis. However, self-diagnose by wearable differs from self-diagnosis using the information found online, as it is backed up with data understood as objective; this is contrasted with the dependency solely on the person’s subjective feeling fitting their bodily feelings to the condition described online. Wearables can give the impression that they are more reliable. In this sense, wearables also affect the evidence that a person needs to have in order to ask for medical help (feeling unwell, deviation in data, or both).

Another fitting process can take place between the first-person and the social perspectives, especially in the case of parties interested in the financial success of wearables. Like any other commercial product, the first-person perspective can be shaped by the branding strategies of wearables companies (Owens and Cribb, 2019). This fitting process affects one’s needs, for example by creating a new need to track and measure activities and functions that one did not even consider tracking and measuring before (and is now willing to do so regardless of a new biomedical need, such as a change in one’s health state or entry to a new risk group). In their discussion on autonomy of users using wearables, philosophers John Owens & Alan Cribb (2019) highlight their concern about the potential harm of these technologies on users’ substantive relational autonomy^23^, for example their potential to manipulate users’ decisions and behaviors for the benefit of corporations. Similarly, Svenaeus (2023) argues that sharing health data with other users and companies commodifying the surveillance bodies (Owens & Alan Cribb 2019, pp. 146–147). These changes in the first-person perspective can eventually create a new normal for society at large. This might also lead to more epistemic injustice, despite the role of the user as the diagnoser.

### Social perspective and bioscientific knowledge

Wearables also extend the social engagement in the diagnosis. The sociologist Deborah Lupton (2014) describes the social communities that are growing in the era of electronic health, where the use of wearables is gaining popularity. The use of wearables produces health data that is then shared online with other users. The community of users exchanges and compares their data and their embodied experiences of measuring themselves. In some cases, the users of these communities also share their coping techniques which helped them to manage their malady^24^, and forming epistemic communities. Some of these emerging social networks, such as Fitbit^25^ and Muse EEG headband (that acts as a personal meditation coach), have similarities to gaming platforms, where users compete against themselves or each other, to gain better measurements or achieve some level of activity that they then can share. This is especially the case when it comes to fitness measurements (see for example: Maturo and Moretti 2018; Whitson 2014).

Through such networks, wearables encourage social engagement between people regarding their health data. The emergence of new social networks that focus on health and the extensive sharing of data that individuals were not used to share before, creates a new normal standard within a specific community, which is not always equivalent to the biomedical normal standard. Deviation from the norm can lead to a diagnosis, where the person is marked as unhealthy or unfit. Basing this new normal on data that is considered scientific knowledge can blur the boundaries between collective knowledge and scientific knowledge.

While bioscientific knowledge like genetic tests are already been used in social media to promote racist agendas (see, for example: Mittos et al. 2020), the new normal created by the use of wearables does not necessarily driven by ideology or political aware intentions. Nevertheless, it can be affected by lack of trust between patients and medical institutions (Goldenberg 2021; Jasanoff et al. 2021), misinformation, or simply follow the dynamic of the power structure and epistemic status in a specific group. These diagnosis practices are already taking place in contexts at the periphery of health, such as work environments and sport education, where workers or students are obliged to use wearables to track their measurements, body language (such as posture and tone of voice) and habits, so their employers and teachers can get an overview of their performance (Lupton 2014; Owens and Cribb 2019). That is to say, the new normal emerging from the social perspective by the use of wearables can affect all aspects of life, and therefore contributes to the growing medicalization.

The focus on self-responsibility in health management promoted by wearables can also lead to new stigmas on sick individuals who do not conduct a responsible, healthy lifestyle according to the new emerging standards of wearable users (Hofmann and Svenaeus 2018). According to Kukla (2022a, p. 318), “dominant and hegemonic groups control conceptual resources,” so the concept of health (and by that also malady) can be used against subgroups in a society. That is to say, dominant and hegemonic groups can control not only what is regarded as sickness (collective knowledge) but also, through data produced by wearables, they can point to the diagnosis of a biomedical condition, i.e., disease. As such, we can anticipate less epistemic justice for marginalized groups despite increased social engagement in diagnostic processes.

## Concluding remarks

The biomedical, first-person, and social perspectives on diagnosis can conflict in determining whether someone is healthy or not; however, while these three perspectives are responsible for diagnosing three different aspects of health— bioscientific, individual, and collective —in the case of wearables they intermingle as they all aim at diagnosing a disease (bioscientific knowledge). The users, as individuals and as communities, are asked to embrace and act upon the biomarkers. So, wearables create new dynamics, in which the three perspectives can indicate not only different health states but also conflicting perspectives regarding the existence of a biomedical condition. Diagnosis of a disease is no longer made exclusively by biomedical professionals (perspective), and what counts as biomedically pathological (aspects) is not exclusively (or almost exclusively) determined by the biomedical community but affected and shaped by the algorithms in use and commercial interests. In addition, wearables generate a possible conflict between what the data produced by the wearables indicate and the clinician’s perspective. In other words, the diagnosis of a disease can be made without the biomedical community perspective.

A higher engagement of individuals and communities in diagnostic processes is important to achieve better medical knowledge and reduce epistemic injustice. However, it is also important that the representation of these perspectives will be genuine so that the knowledge they carry will be shared. It is, therefore, essential to pay attention to the dynamics between aspects and perspectives, what knowledge is produced by which perspective, and on which basis. It is also important to be attentive to fitting processes between aspects and perspectives; while these fitting processes have the potential to bridge mismatches between disease, illness, and sickness, they can also distort the way one feels and connects to one’s own body and create an illusion of autonomy and participation. Wearables are, therefore, a double-edged sword: by using the binocular model, I showed that while wearables promote more participation that has the potential to contribute to different diagnostic processes that can improve health and well-being, they mainly promote a multi-diagnosis of a disease, which limits the different actors’ ability to contribute their knowledge and, therefore, restricts health promotion. By conducting conceptual scaffolding, I showed that diagnosis is made through an epistemic division of labor and that wearables are changing the dynamics of this division of labor in ways that require our attention.

## Footnotes

1 I thank Gry Oftedal for this suggestion.

2 See especially Boorse (2011), Nordenfelt (1995), and Wakefield (2001) who employed this methodology and according to Lemoine (2013) affected the discourse that followed them.

3 According to Belleri, cognitive and theoretical functions enable categorization and facilitate reasoning, prediction, and explanation. Practical functions involve non-evaluative judgments, decision-making, action-guiding and inter-subjective coordination (Belleri 2021, p. 5).

4 This aligns with and extends the motivation to understand the context of scientific practice more generally, which it shares with feminist theory of science (see for example, Longino 1990).

5 Here, I do not sharply differentiate between biomedical research and practice. However, it is important to note that biomedical practice is dependent on the bureaucratic structure of the healthcare system in a specific social context (including legal and political factors), so any given biomedical perspective is partially constructed, and constrained, by specific collective aspects.

6 The use of ‘individual’ here does not imply ‘biological individuality’. I could have used the term ‘personal’ or ‘private’; however, these terms are associated with subjectivity, and I therefore find them confusing.

7 I use ‘first person’ instead of ‘patient’ perspective, as in the triad model, to indicate that a person does not have to be regarded as a patient in practice. First-person does not imply here that the model is necessarily anthropocentric.

8 The individual can also have access to knowledge about herself that is not accessible to others. This knowledge is embedded in her perspective.

9 A similar approach can be found in the new BPS model by Bolton and Gillett (2019) and in recent theoretical approaches to BPS in psychiatry, see for example McConnell (2020). However, in both cases the first-person perspective is still defined in terms of aspects.

10 Following Bschir and Lohse (2022), a democratic society that takes into consideration different knowledge resources can perform an interdisciplinarity pluralism.

11 This collective aspect can cause one to be morally vulnerable and lose confidence in the world (so it affects the first-person perspective), which in turn can affect both individual and bioscientific aspects, as in the case of post-trauma (see: Corbi 2017).

12 These issues are of special interest to the philosophy of psychiatry but are equally relevant to somatic diagnoses.

13 Etymologically, ‘bi’ is also related to the ‘dia’ (“Diagnosis,” n.d.).

14 On the individual level, sometimes we can talk about an individual who holds the three perspectives: a doctor who is also a patient and a member of the society in focus.

15 As explained in part 1, these three concepts traditionally have additional layers so that disease can be conceived as a biomedical (and mental) phenomenon, which is objectively observed, examined, or empirically measured (or in principle could be), while illness is a phenomenon understood as a personal experience, and sickness is a phenomenon of social convention (see Hofmann 2016).

16 I thank Stefano Canali for this point. A nuanced discussion on P-medicine and wearables is a subject for a separate article.

17 In addition, as Canali et al. (2022) highlight, there is a use of different measuring methods in different wearables which makes it difficult to compare data on the same variable when using different wearables. In their paper, they give examples of saturation biomarkers that are measured using different sensors or inferential methods, which generate different results.

18 While health care professionals currently lack engagement in social media platforms in their work place this attitude towards these platforms can change as the benefits of their involvement in digital communities (and in that also in those associated with wearables) becoming more evident (see, for example: Sendra et al. 2023).

19 Wearables may provide a fair tracking process. Still, the living conditions of users could be very different, as could their ability to interpret the data and change their habits (Owens and Cribb 2019). In other words, there is not necessarily equality between users in the social context that would allow them to manage their health under the same conditions.

20 It is essential to distinguish between cases in which the patient is also monitored by healthcare professionals, who obtain the data and contact the patient if they diagnose a problem, and cases in which the *responsibility* for tracking and interpretation is *solely on the user*. In the latter kind of case, the user’s role as a diagnoser (first-person perspective) is more prominent.

21 Wearables might also influence the doctor-patient relationship and by that affect epistemic injustice. They can potentially reduce healthcare costs by early detection and prevention of some conditions, and by giving users more responsibility for managing their own health, they can save time in the clinic. However, the direction in which these changes will direct doctor-patient relationship is still an open question. Whether it will make them closer or create more distance depends on the social, economic, and bureaucratic conditions in a given healthcare setting.

22 For example, “a patient can feel his “large intestines a bit bound” based on seeing X-ray images only” (Nessa and Malterud, 1998, as cited in: Hofmann and Svenaeus 2018, p. 4).

23 Similarly, the political scientist Barbara Prainsack (2017, pp. 189–192) distinguishes between four models of empowerment.

24 In her book “Care at a Distance: On the Closeness of Technology”, the anthropologist Jeannette Pols (2012) describes a case of a patient with progressive lung disease COPD who uses a mobile device that measures her oxygen saturation level in the blood and a webcam that is used to talk with her carer and with friends dealing with COPD. Although the webcam is not a wearable device, the community built up through it helps the patient to share experiences and knowledge about their condition and their self-monitoring.

25 Google, which recently bought Fitbit, shut down fitness gamification features like Fitbit Challenges and Adventures.
