# Correction: Conceptual scaffolding for the philosophy of medicine

**DOI:** 10.1007/s11019-024-10242-7

**Published:** 2024-12-20

**Authors:** Yael Friedman

**Affiliations:** The Centre for Philosophy and the Sciences (CPS), Department of Philosophy, Classics, History of Art and Ideas, University of Oslo, Oslo, Norway

**Medicine, Health Care and Philosophy**

10.1007/s11019-024-10231-w

In this article, the following paragraphs have been modified for comprehensibility post publication.

Under section ‘Conceptual scaffolding of malady: two ancestor models’, the last line of 4th paragraph should read as “… specific discipline (I will develop this idea in the section titled “The binocular model of plural medicine”).” Instead of “… specific discipline (I will develop this idea in Sect. 5).”

In the last paragraph of section ‘Conceptual scaffolding of malady: two ancestor models’, the last sentence should read as “The binocular model, presented below, is therefore not …” instead of “The binocular model, presented in Sect. 5, is therefore not …”

In the Introduction section of Part 2, the second paragraph should read as “In the next sections I will introduce the concept of diagnosis and health wearable technologies. The section titled “Wearables and the concept of diagnosis” is dedicated to the conceptual scaffolding. The last section concludes by showing how the division of labor between the different actors in the medical realm is changing due to the implementation of wearables, and what are the possible epistemic implications of such a change.” instead of “In the next sections I will introduce the concept of diagnosis (Sect. 2) and health wearable technologies (Sect. 3). Section 4 is dedicated to the conceptual scaffolding. Section 5 concludes by showing how the division of labor between the different actors in the medical realm is changing due to the implementation of wearables, and what are the possible epistemic implications of such a change.”

The original article has been corrected.

